# Seroconversion after needlestick injuries – analyses of statutory accident insurance claims in Germany

**DOI:** 10.3205/dgkh000311

**Published:** 2018-07-06

**Authors:** Madeleine Dulon, Dana Wendeler, Albert Nienhaus

**Affiliations:** 1 Institution for Statutory Accident Insurance and Prevention in the Health and Welfare Services (BGW); 2 University Medical Center Hamburg-Eppendorf (UKE), Institute for Health Services Research in Dermatology and Nursing (CVcare)

**Keywords:** seroconversion, needlestick injury, medical personnel

## Abstract

**Objective:** After a needlestick injury (NSI) with contaminated blood, there is a risk of seroconversion. Statutory accident insurance (SAI) claims data were used to determine the numbers of seroconversions for hepatitis B and C viruses (HBV, HCV) and for HIV.

**Materials and methods:** Cases of HBV, HCV or HIV infection recognised as occupational diseases between 2006 and 2015 were selected from the BGW (Berufsgenossenschaft für Gesundheitsdienst und Wohlfahrtspflege) database. Cases where an NSI was reported to the accident insurer before the diagnosis of the infectious disease was made were included in the analysis. The causal link between the infection and the NSI identified was estimated based on diagnostic findings in medical case files.

**Results:** In total, 566 cases with an occupation-related HBV, HCV or HIV infection were identified, including 44 cases where an NSI had been reported before diagnosis. Data on file indicated a possible causal link in 34 cases. In 16 of the 34 cases, seroconversion after the NSI was proven by diagnostic findings; in 13 of the 34 cases, seroconversion was possible but not proven because of the lack of initial findings. The index case was known in 23 of the 34 cases. The injuries occurred most often during waste disposal and high-risk procedures such as taking blood samples. The injuries were most often caused by cannulas for intravenous puncture. Subcutaneous devices were involved in two NSIs but there was no information on the initial serology or known index case.

**Conclusions:** It is possible to identify seroconversion in SAI claims data. However, data on the injured person’s initial infection status is often incomplete and this makes it difficult to assess any causal link. The incidence of seroconversions resulting from injuries from subcutaneously applied devices is apparently low; this is consistent with the literature.

## Zusammenfassung

**Ziel:** Nach einer Nadelstichverletzung (NSV) mit kontaminiertem Blut besteht das Risiko einer Serokonversion. Um die Zahl der Serokonversionen durch Hepatitis B- und Hepatitis C-Viren (HBV, HCV) sowie HIV zu bestimmen, wurden die Routinedaten der Berufsgenossenschaft für Gesundheitsdienst und Wohlfahrtspflege ausgewertet.

**Methoden:** Die Auswertung basiert auf Versicherten, deren HBV-, HCV- oder HIV-Infektion zwischen 2006 und 2015 als Berufskrankheit (BK) anerkannt wurde, und von denen vor der Diagnosestellung eine NSV gemeldet wurde. Eine Einschätzung des Ursachenzusammenhangs zwischen NSV und Infektion wurde anhand von Befunden aus den Unfall- und BK-Akten vorgenommen.

**Ergebnisse:** Bei 566 Versicherten wurde zwischen 2006 und 2015 eine Infektionserkrankung mit HBV, HCV und HIV als BK anerkannt. Darunter waren 44 Versicherte, bei denen ein Arbeitsunfall im Zusammenhang mit einer NSV gemeldet wurde. Nach Aktenlage war bei 34 Fällen ein Ursachenzusammenhang möglich. Nach der NSV ist es in 16 der 34 Fälle zu einer Serokonversion gekommen; in 13 der 34 Fälle ist eine Serokonversion möglich aber nicht belegt wegen fehlender Ausgangsbefunde. Der Indexpatient war in 23 der 34 Fälle bekannt, davon in 14 Fällen auch der Infektionsstatus. Die Unfälle ereigneten sich am häufigsten bei der Entsorgung und bei risikobehafteten Prozeduren wie der Blutentnahme und wurden am häufigsten durch Kanülen für intravenöse Punktionen verursacht. An zwei NSV waren subkutan verwendete Instrumente beteiligt (Insulinpen und Lanzette). Zu beiden Fällen lagen keine Angaben zum serologischen Ausgangsbefund oder Indexpatient vor.

**Schlussfolgerungen:** Die Identifizierung von Serokonversionen in Routinedaten der Gesetzlichen Unfallversicherung ist möglich. Allerdings sind die Daten zum Ausgangsbefund des Verletzten und zum Infektionsstatus der Indexperson oftmals unvollständig, was die Beurteilung eines Zusammenhangs erschwert. In Übereinstimmung mit der Literatur scheint der Anteil an Serokonversionen durch Verletzung an subkutan verwendeten Instrumenten gering zu sein.

## Background

Occupational needlestick and sharps injuries (NSIs) continue to pose a significant hazard for healthcare workers (HCWs). Frequent exposure to NSIs is a major health concern, due to the associated risk of acquiring hepatitis B, hepatitis C and HIV. It has been estimated that about 40% of hepatitis B and hepatitis C infections and 2.5% of HIV infections in HCWs are due to sharps injuries [1]. To reduce the risk of injury and infection, various measures have been implemented during the last 20 years, such as the Needlestick Prevention and Safety Act in 2000 and European Union Council Directive 2010/32/EU in 2010 [2], [3]. This Directive was implemented in Germany via an amendment to the Ordinance on Biological Agents (BioStoffV) and in the specification of requirements in the Technical Regulations for Biological Agents (TRBA 250) [4]. The most commonly reported healthcare-related sharps injuries were associated with venepuncture [5]. The magnitude of the risk from devices for subcutaneous or intramuscular injection is currently under discussion. The WHO recommends the use of safety engineered devices for intramuscular and subcutaneous injections, although the evidence for this recommendation is weak regarding the risk of seroconversion [6], [7].

The risk of transmission depends on several factors, including type of injury, transferred blood volume, viral load of the source patient, immune status of the recipient, and risk reduction strategies implemented in the healthcare setting [5]. Several studies have examined the risk of seroconversion after an NSI [8], [9], [10], [11], [12], [13]. According to a recently published study, the seroconversion rate for the hepatitis C virus (HCV) was 0.1%; a literature review included in the study showed rates between 0.0% and 10.0% for HCV seroconversion [11]. The authors of a study on HIV seroconversion reported that no seroconversion was observed during a follow-up of 266 HCWs after exposure to HIV-contaminated body fluids, and a literature review included in the study showed rates between 0.0% and 1.5% for HIV seroconversion [14]. 

In Germany, NSIs are not routinely monitored. In about the year 2000, it was estimated that up to 500,000 NSIs per year occurred in Germany, although the reporting rate is estimated to be low, approximately less than 50% [15]. The majority of the NSIs are reported because the costs of medical treatment will be covered by the accident insurance provider. Analyses of the claims data of a single insurer, the Institution for Statutory Accident Insurance and Prevention in the Health and Welfare Services (the BGW), showed that the number of reported NSIs increased between 2007 and 2016 by 41.7%, from 37,429 to 53,027 [16]. 

The aim of the present study was to investigate whether statutory accident insurance (SAI) claims data can be used to identify cases in which there was a causal link between an NSI and an HBV, HCV or HIV infection. Our second objective was to examine to what extent devices for subcutaneous or intramuscular injection were involved in seroconversion. 

## Materials and methods

### Database

This analysis is based on the SAI claims data of the BGW. SAI claims data are administrative data routinely recorded on notification of accidents at work or occupational diseases. SAI claims data do not contain any information on the course of the accident or on diagnostic findings. As this data is needed in order to decide whether a causal link exists between the occupational infectious disease (OID) and the identified NSI, it was recorded by claim administrators in the case files and added to the dataset subsequently. 

### Reporting of occupational diseases 

In Germany, physicians are obliged by law to report suspected claims of occupational disease to the relevant accident insurance provider. The insurance provider has to determine whether liability exists. If a relationship between the activity at work and the occupational disease is likely and if German legal stipulations are fulfilled, the claim is recognised [17]. Claims without a clear link to occupational exposure are rejected. Occupational disease BK-No. 3101 in Germany includes infectious diseases that can be transmitted from person to person but can only be recognised as occupational diseases if caused by activities in the health service or in areas with comparable infection risk (The German Code of Social Law VII §9) [17]. Often, possible exposure to infection-causing pathogens cannot be established retrospectively and the timepoint of pathogen transmission may not be verifiable, especially if infections are initially asymptomatic. In this case, the burden of proof may be eased for the insured person by recognising an infectious disease in the sense of BK-No. 3101 as an occupational disease, if the requirements are fulfilled and there is no evidence for causality outside the insured task [18]. 

### Data selection

In our study, seroconversion was defined as conversion from negative test results at the time of the NSI to positive results during the follow-up. As there is no specific code for NSIs in the SAI claims data, an NSI was defined as an accident which was documented as a percutaneous injury to the hand with additional post-exposure measures such as blood tests.

Data collection was carried out on 26 January 2016 for claims fulfilling the following inclusion criteria:

- An OID caused by HBV, HCV or HIV and recognised between 2006 and 2015
- An NSI reported for the identified claim
- The date of the NSI preceded the date of the diagnosis
- The interval between the date of the NSI and the diagnosis was no more than 12 months, or twice the incubation period, in order to reduce the chance of alternative exposures.

Information for the following variables was collected from the paper-based files: date and serological findings of the initial and follow-up examinations, index case (known and serological findings), device involved, and activity during which the NSI was suffered. 

Finally, on the basis of the NSI and the OID files, the claims administrator was requested to evaluate whether there was a causal link between the OID and the NSI identified in the SAI claims data. The possible answers were likely, possibly and unlikely to be linked. Further comments on the claim were possible. 

## Results

**Figure 1:**
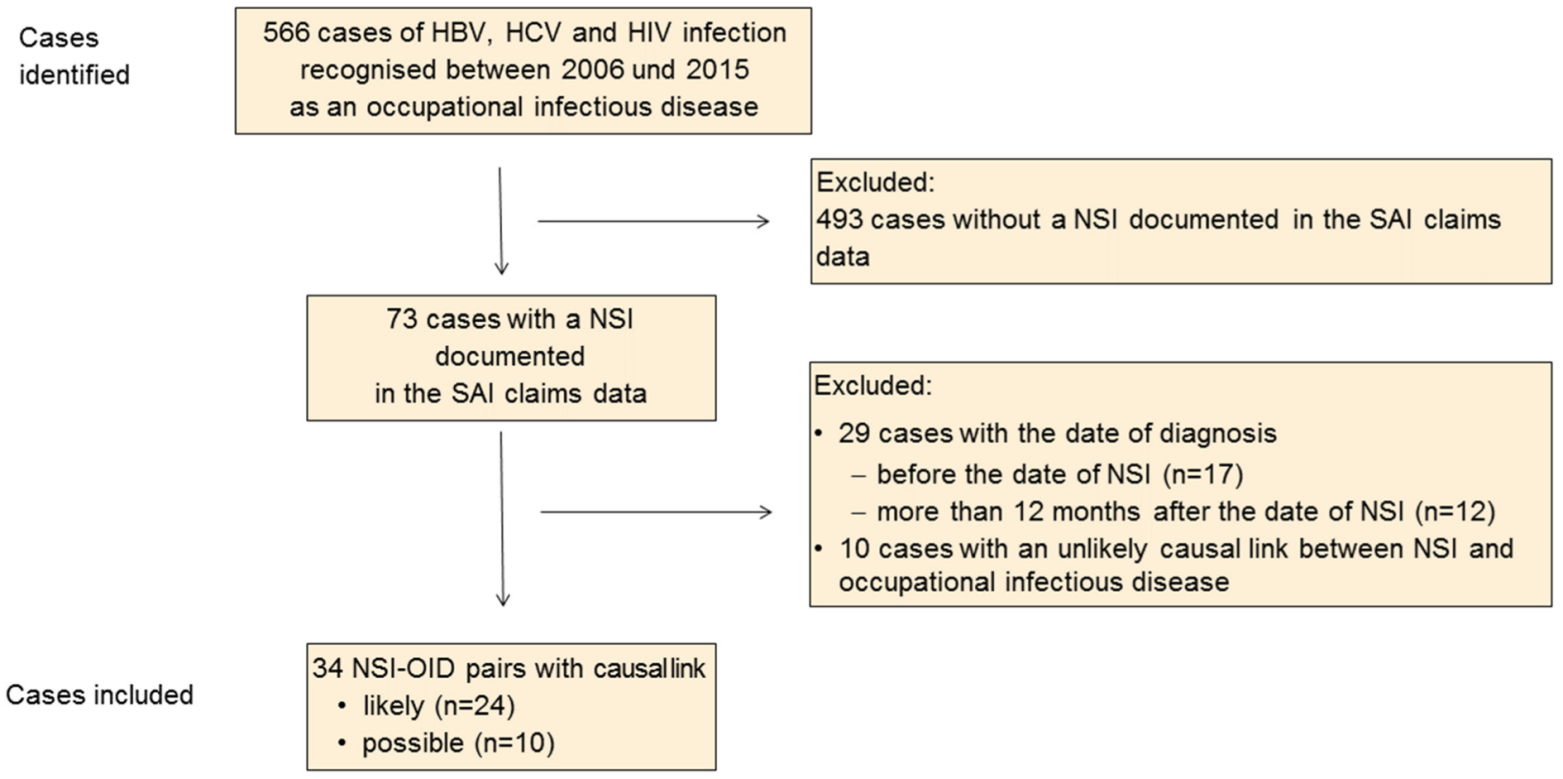
Flow chart for included and excluded cases; NSI, needlestick injury; OID, occupational infectious disease.

Within a period of 10 years, 566 suspected claims involving HBV, HCV and HIV were recognised as OID. These included 73 cases in which an NSI was identified and recorded in the SAI claims data (Figure 1 (Fig. 1)). Twenty-nine cases were excluded because the date of the diagnosis was either before the date of the NSI (n=17) or more than 12 months after the date of the NSI (n=12). Another 10 cases with an unlikely link between the infectious disease and the NSI identified were excluded.

Of the 34 NSI-OID pairs identified from the SAI data, 24 (70.6%) exhibited a likely causal link, and for 10 no conclusion could be drawn; for these cases it is assumed that a causal link is possible in principle (Table 1 (Tab. 1)). 

At the point of the initial test, the serological findings for the 34 NSI-OID pairs were negative in 16 cases (47.1%), positive in five cases (14.7%) and unknown (without initial findings) in 13 cases (Table 2 (Tab. 2)). The claim administrators’ comments show that – regardless of serological status at the time of the NSI – the burden of proof was eased for 18 (52.9%) of the 34 NSI-OID pairs.

NSIs occurred most frequently during the disposal of cannulas or while clearing up, or were associated with high-risk procedures (taking blood samples, insertion or removal of catheters) (Table 3 (Tab. 3)). NSIs were most frequently caused by intravenous catheters. In two injuries a subcutaneous device was involved – an insulin pen and a lancet. In these two cases, there were no findings on the serological status before the NSI and the index case was unknown. The index case was known for 23 claims, of which the infection status was known for 14 index cases.

## Discussion

This is the first attempt to determine the frequency of seroconversion after NSI on the basis of statutory accident insurance claims data. Over a period of 10 years, a total of 24 claims were identified in which the causal link between the NSI and the HBV, HCV or HIV disease was assessed as likely and a further 10 claims as possible in principle. The serological findings indicate that seroconversion was likely in 16 cases. As there is no information on the genotype, it is not possible to draw a reliable conclusion about the causal link. In 13 cases, seroconversion is possible but not proven, as there were no findings on the status prior to the NSI. In these 13 cases, it was apparently not possible for the claim administrators to assess whether the particular NSI was the cause of infection or the risk of infection was increased in general and the burden of proof was eased for the claim. Prior infections were responsible for the positive initial findings in two HBV and three HCV cases. 

Thus, it is possible in principle to use SAI data to identify cases in which seroconversion took place after an NSI, if findings from accident and occupational disease files are considered. As only 34 cases were identified in the BGW database between 2006 and 2015, while the average number of NSIs reported annually to the BGW within the same period was around 44,000, the seroconversion rate appears to be low. The identification of seroconversion rates on the basis of SAI claims data is made more difficult by the fact that information on NSI or blood contact was only documented in a small proportion (n=72) of the 566 cases of HBV, HCV or HIV infections recognised as occupation-related. For the other 494 recognised claims, occupational activity associated with an increased risk of infection was presumed. The rules on presumption state that an OID may be recognised in such cases even though the infection route cannot be precisely identified. 

The risk of seroconversion after an NSI is particularly dependent on the type of injury and the quantity of blood transferred [19]. The seroconversion risk is thought to be particularly high when hollow-bore needles are used for venous procedures [9], [19]. Studies on the risk of seroconversion rarely contain data of the device involved in the NSI [11], [13]. A few case reports and seroconversion case series provide more details about the circumstances of NSIs [9], [20], [21]. Cannulas for subcutaneous injection are rarely mentioned in relation to the risks of seroconversion. Case reports from France describe 12 well documented cases involving subcutaneous or intramuscular cannulas and sharp devices such as sewing needles or lancets [21]. In our datasets, subcutaneous devices were only involved in two of the 34 NSIs identified. We cannot draw any conclusions about a causal link in these two cases, as essential information such as the status before the NSI or information on the index case was missing. As cannulas for subcutaneous injection are one of the most common causes of NSIs, one would have expected subcutaneous devices to have been mentioned more often as being involved in seroconversions after NSIs [22]. 

### Strengths and weaknesses 

The data used are claims data collected during statutory accident insurance administration processes, but the depth of the information is limited. This was the first attempt to link accident data with occupational disease data. This linkage depends on several factors. Both accident and occupational disease datasets need to be filled. Therefore, some linked cases might have been missed. However, this underreporting is most probably independent of the kind of device responsible for the accident. 

## Conclusions

Routine statutory accident insurance data are a possible source of data to estimate the risk of seroconversion after NSI. In the present study, information on the course of the accident was only supplied in two-thirds of cases. Cannulas used for venous procedures are thought to pose a high risk of seroconversion and were the devices most often involved in NSIs in our study. In accordance with the published literature, the risk of seroconversion after injury from subcutaneous devices appears to be slight. 

## Notes

### Competing interests

All authors are employed by the BGW. However, neither the Board of Directors nor the Board of Self-Government of the BGW had any influence on the subject matter of the study, data collection, data analysis or data interpretation. The authors declare that this affiliation gives rise to no conflict of interests.

## Figures and Tables

**Table 1:**
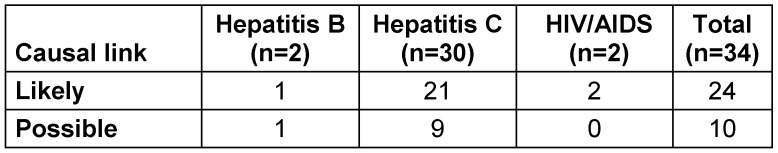
Assessment of the causal link between needlestick injury and occupational infectious disease by disease

**Table 2:**
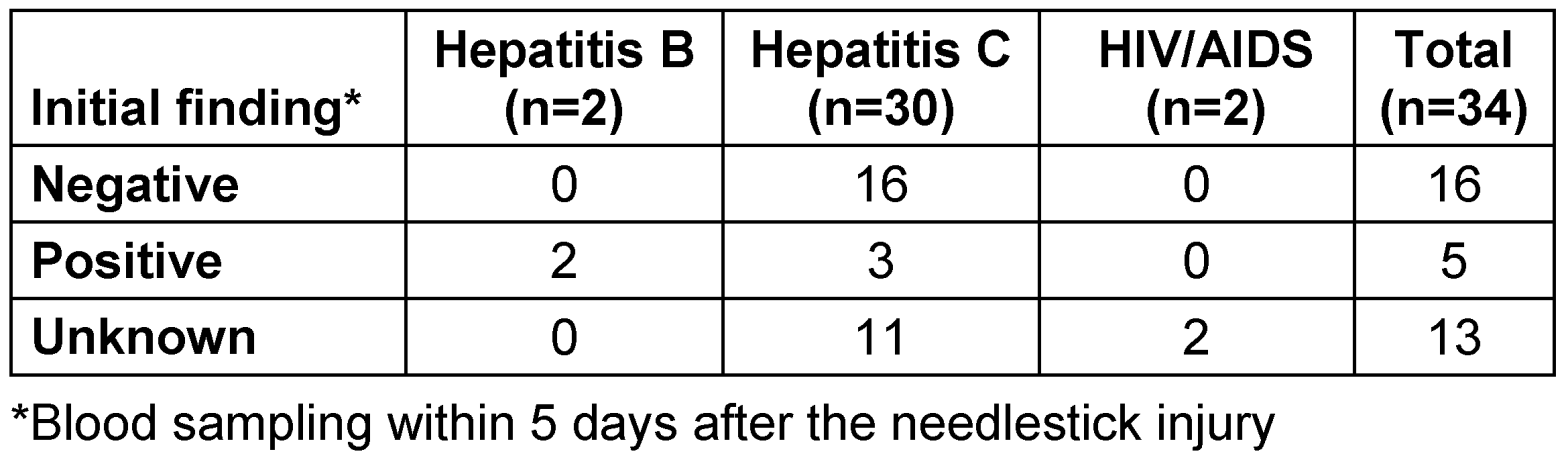
Serological finding of the initial test by disease

**Table 3:**
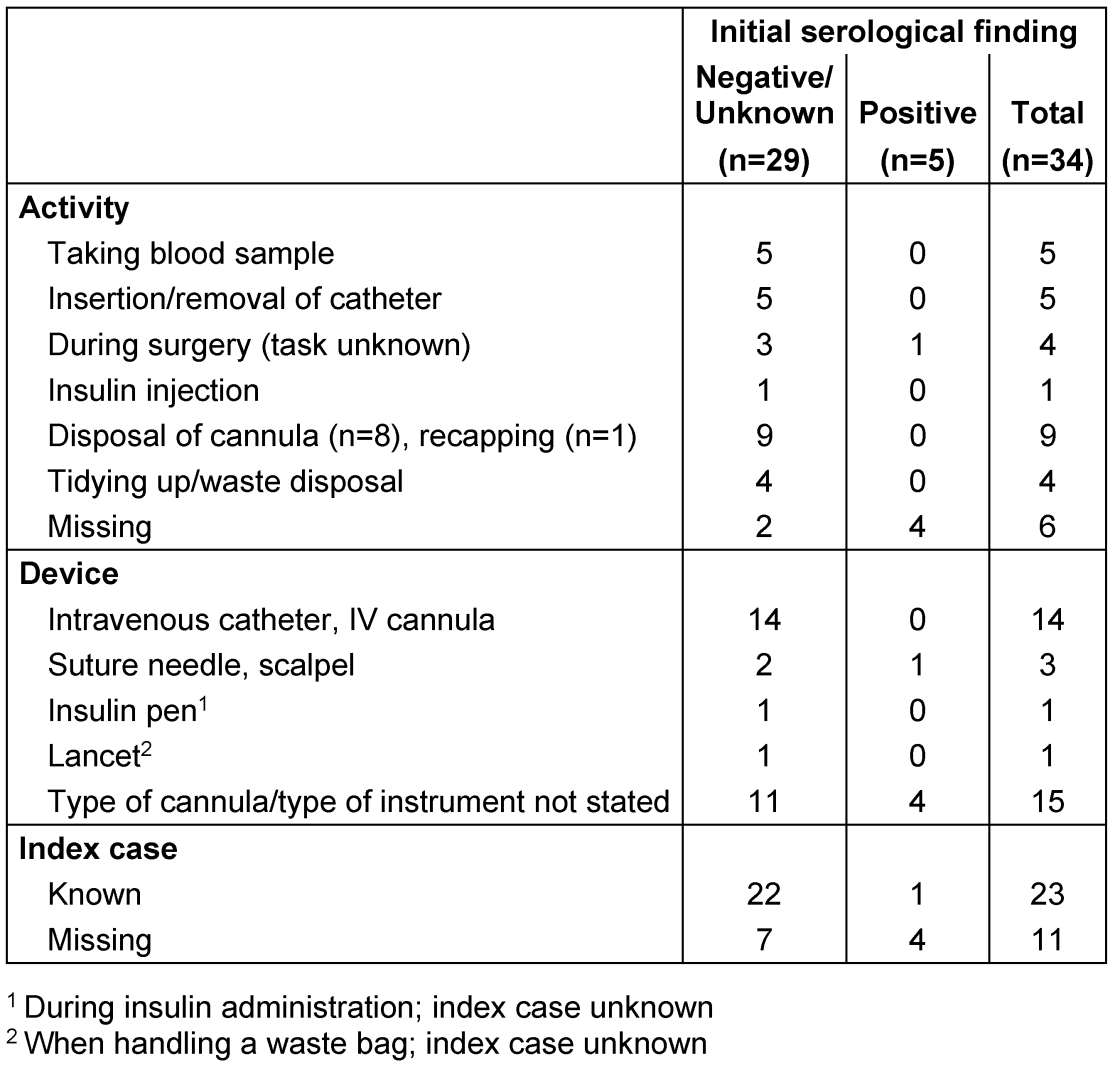
Activity and device involved in NSIs and index case by initial serological finding (NSI, needlestick injury)
